# Real-time augmented reality navigation in brain glioma resection: a pilot feasibility study

**DOI:** 10.3389/fsurg.2026.1802289

**Published:** 2026-05-28

**Authors:** Peihai Zhang, Kai Zhang, Huiting Liu, Xuejun Yang

**Affiliations:** 1 Beijing Tsinghua Changgung Hospital, School of Clinical Medicine, Tsinghua Medicine, Tsinghua University, Beijing, China; 2 Peking Union Medical College Hospital, Beijing, China

**Keywords:** augmented reality, brain glioma, HoloLens 2, navigation, surgery

## Abstract

**Objective:**

To evaluate the clinical feasibility and technical performance of augmented reality (AR) navigation for brain glioma resection in a preliminary patient series.

**Methods:**

From April 2024 to May 2025, 6 patients with brain glioma were enrolled in this pilot study. Preoperative magnetic resonance imaging (MRI), diffusion tensor imaging (DTI), and magnetic resonance angiography (MRA) data were processed and imported into an AR head-mounted device. The 3D holographic models were reconstructed to enable preoperative surgical planning, simulation of resection pathways, and intraoperative AR navigation. The primary outcomes included AR registration time, target registration error (TRE) verified by an optical navigation system (StealthStation S7), extent of resection on postoperative MRI, and neurological function assessed by the Modified Rankin Scale (mRS) and detailed neurological examination at 3 months.

**Results:**

Gross total resection of the contrast-enhancing tumor was achieved in all patients. The mean AR registration time was 4.7 ± 0.7 min. The surface-matching registration yielded a TRE of 2.1 ± 0.4 mm compared to the StealthStation reference. Postoperative mRS scores improved significantly at 3 months (*p* = 0.03).

**Conclusion:**

This pilot study demonstrates that AR navigation is technically feasible for brain glioma resection. However, the small sample size limits conclusions about clinical efficacy.

## Introduction

During resection of gliomas, neurosurgeons face the challenge of maximizing tumor removal while preserving neurological function (1, 2). Key technical challenges include accurate preoperative planning, intraoperative delineation of tumor boundaries and eloquent areas, and real-time guidance to avoid critical structures (3, 4).

Augmented reality (AR) integrates virtual data into the real surgical field by superimposing 3D holographic models onto the patient's anatomy. This technology has gained interest in neurosurgery because it may reduce cognitive load and the need to shift attention to external screens (5–8). Previous reports have described the use of AR for various cranial procedures, including tumor resection, ventricular drain placement, and spine surgery (9–15). However, most studies remain small and lack quantitative accuracy assessment or rigorous outcome evaluation.

Building on our preliminary experience with AR-guided procedures (16–18), we conducted a pilot study to assess the feasibility, registration accuracy, and initial clinical results of AR navigation in six patients undergoing brain glioma resection. The objective was to gather technical data and identify potential benefits and limitations before designing larger comparative trials.

## Methods

Between April 2024 and May 2025, 6 patients with suspected brain glioma scheduled for microsurgical resection were enrolled in this prospective pilot study. The study was approved by the institutional review board of Beijing Tsinghua Changgung Hospital. Written informed consent was obtained from all patients. Exclusion criteria included contraindications to MRI, inability to tolerate surgery, or prior cranial surgery that could interfere with image registration.

All patients underwent preoperative MRI, DTI, and MRA. The acquired DICOM data were processed using 3D Slicer, DSI Studio, and the SurgicalAR workstation to generate multimodal fusion-based 3D holographic models. These models included the tumor (enhancing and non-enhancing components), peritumoral edema, major blood vessels, and corticospinal tracts derived from DTI.

The SurgicalAR platform was used with a HoloLens 2 head-mounted display (Microsoft, Redmond, USA). During surgery, the patient's head was fixed in a Mayfield clamp, and the reference frame of an optical navigation system (StealthStation S7, Medtronic, Minneapolis, USA) was attached to the clamp. Point-cloud data of the facial surface were acquired using a navigation probe, and surface-matching registration was performed to align the holographic model with the patient. The HoloLens 2 sensors tracked the surgeon's head position relative to the reference frame, updating the hologram in real time (Supplementary Video 1).

The surface-matching registration in SurgicalAR employs an iterative closest point (ICP) algorithm. A standard navigation probe (tracked by the StealthStation S7) was used to digitize approximately 150–200 points distributed over the patient's facial surface, including the forehead, orbital rims, and nasal bridge. The acquired point cloud was transmitted to the SurgicalAR workstation, where it was matched to the 3D surface model extracted from preoperative MRI. The ICP algorithm minimized the root-mean-square distance between the point cloud and the surface model.

To quantify registration accuracy, we measured the target registration error (TRE) by touching three anatomical landmarks with the probe and comparing the position indicated by the AR overlay with the actual probe tip. The TRE was defined as the euclidean distance between the virtual and real points.

All surgeries were performed by the same senior neurosurgeon (X.Y.). After registration, the surgeon used the AR display to visualize tumor margins, adjacent vessels, and fiber tracts directly within the operative field. Tumor resection was performed using a standard surgical microscope (Zeiss OPMI PENTERO 900). The AR information was not optically integrated into the microscope's eyepieces; therefore, the HoloLens 2 and the microscope were used in an alternating fashion. For surgical steps that did not require high magnification, such as craniotomy, dural opening, tumor localization before microscopic dissection, and wound closure, the surgeon wore the HoloLens 2 headset and relied on AR navigation as the primary guidance modality. During microsurgical dissection, the surgeon removed the HoloLens 2 and operated exclusively through the microscope to maintain optimal optical quality and ergonomics. If navigation guidance was needed during resection, for example, to confirm tumor margins or the location of critical fiber tracts, the surgeon briefly re-donned the HoloLens 2 to view the AR overlay, then removed it again to resume microsurgical dissection. The StealthStation S7 optical navigation system was used concurrently throughout the procedure for cross-verification of anatomical landmarks.

Postoperative contrast-enhanced MRI was obtained within 48 h to assess the extent of resection. Gross total resection (GTR) was defined as no residual contrast enhancement on T1CE images. Neurological function was evaluated preoperatively and at 3 months postoperatively using the Modified Rankin Scale (mRS) and a structured neurological examination that included assessment of motor strength (MRC, Medical Research Council scale), language (aphasia severity rating), and seizure frequency. The change in mRS was analyzed with the Wilcoxon signed-rank test (SPSS version 26.0). A *p*-value < 0.05 was considered statistically significant.

## Results

6 patients (mean age 62.0 ± 10.3 years) with gliomas in various eloquent locations were included. All tumors were high-grade gliomas (four glioblastomas, two anaplastic astrocytomas). The mean total operative time was 265 ± 28 min, which did not differ significantly from the institutional average for similar cases performed with conventional navigation (260 ± 30 min). No patients experienced postoperative hemorrhage, infarction, or infection.

At 3-month follow-up, all three patients with preoperative epilepsy were seizure-free without medication adjustment. Among the 5 patients with motor or language deficits, 4 showed improvement of at least one grade on the MRC scale or aphasia rating, and 1 patient with severe preoperative motor weakness (MRC grade 2) improved to grade 4. The mean mRS improved from 2.7 ± 1.1 preoperatively to 1.5 ± 0.5 postoperatively (*p* = 0.03, Wilcoxon signed-rank test). Individual mRS changes are shown in Table 1.

**Table 1:** Patient demographics, tumor characteristics, and outcomes.

No.	Age	Location	Pathology	Pre-O neurological deficit	AR registration time (min)	TRE (mm)	mRS	
							Pre-O	Post-O
1	61	Occipital lobe	Anaplastic astrocytoma	Visual impairment	5	2.3	1	1
2	69	Insular lobe	GBM	Epilepsy	4	1.8	2	1
3	45	Frontal and parietal lobe	GBM	Epilepsy, motor disorder	5	2.5	4	2
4	65	Parietal and occipital lobe	Anaplastic astrocytoma	Visual impairment	6	2.1	2	1
5	73	Frontal and temporal lobe	GBM	Epilepsy, aphasia	4	1.9	3	2
6	59	Frontal lobe	GBM	motor disorder	4	2.0	4	2

GBM, glioblastoma; TRE, target registration error; mRS, Modified Rankin Scale.

**Figure 1:**
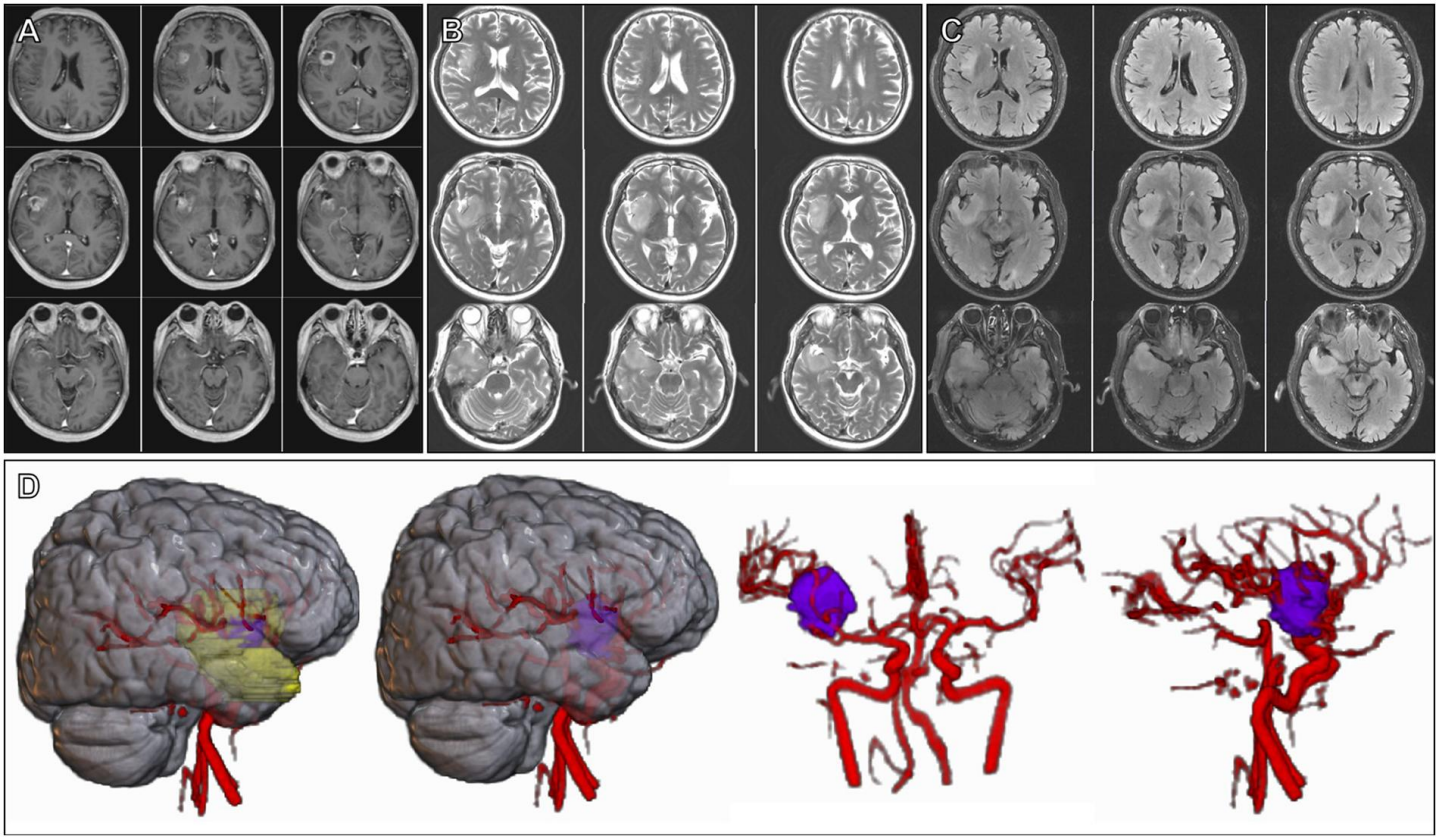
Multimodal image acquisition and 3D model reconstruction. Preoperative MRI indicated a space-occupying lesion in the right insular lobe with significant enhancement [**(A)**, T1CE], and obvious edema areas could be seen around it [**(B)**, T2WI and **(C)**, FLAIR]. Through multimodal image fusion and 3D model reconstruction, the spatial positions of the enhanced part of the lesion (blue) and the non-enhanced part of the lesion (yellow) can be visually located, and the lesion is closely related to the lentiform artery perforation branch of the middle cerebral artery [**(D)**, 3D model].

The preoperative imaging acquisition, and multimodal fused 3D models were successfully completed, which including the basic brain structures, tumors, MRA, and DTI data, as shown in Figure 1.

**Figure 2:**
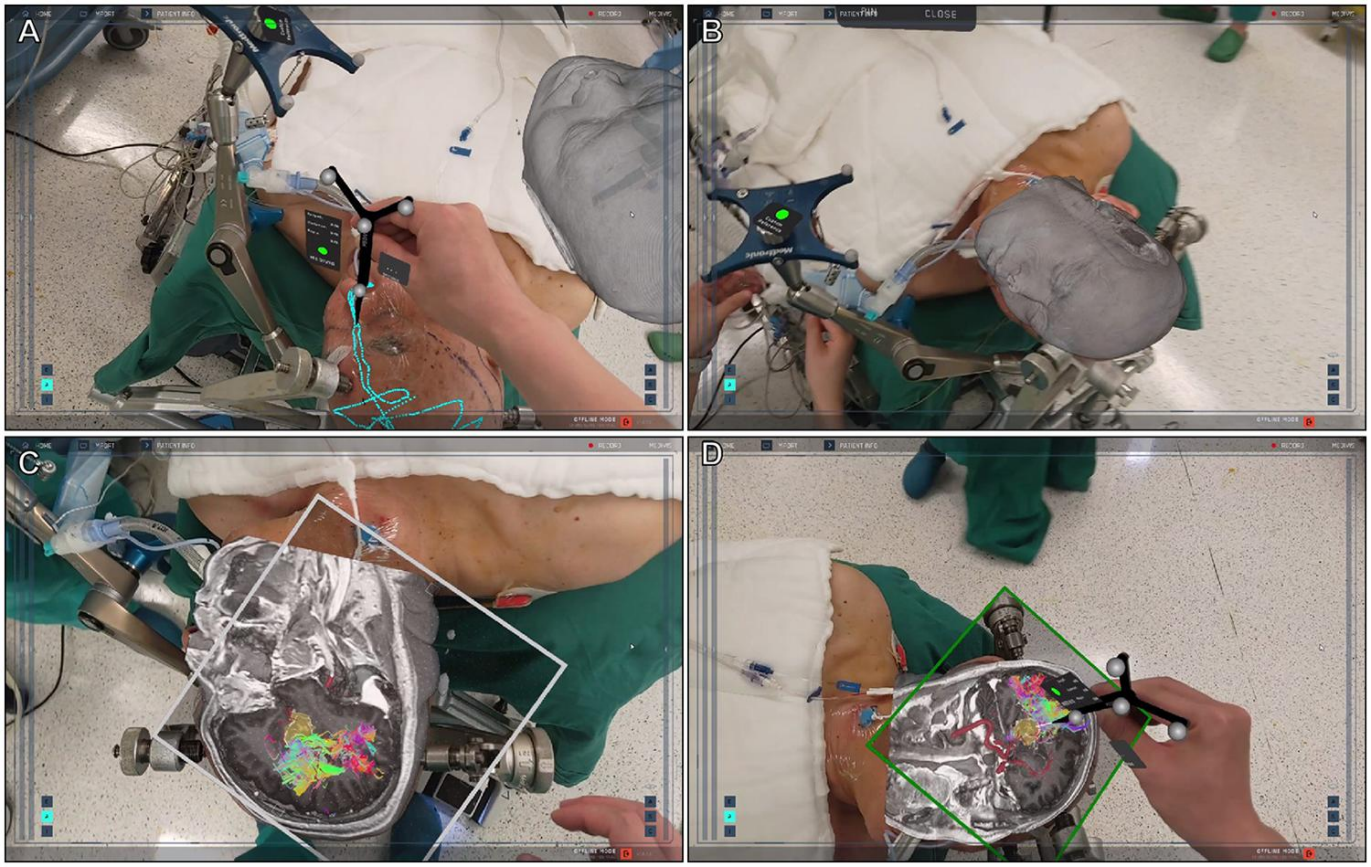
AR navigation registration and virtual surgical simulation. The navigation probe was used to acquire three-dimensional point-cloud data of the patient's head **(A)**. By employing a surface registration algorithm, high-precision *in-situ* overlay of AR image was achieved **(B)**. Under actual surgical positioning, the anatomical location and spatial relationships of the lesion were visualized from different perspectives **(C)**. Using cutting tools, surgical resection was simulated to determine an optimal surgical approach **(D)**.

The virtual surgical simulation was conducted in the actual surgical position to identify critical adjacent functional areas and determine the optimal surgical approach, as illustrated in Figure 2.

**Figure 3:**
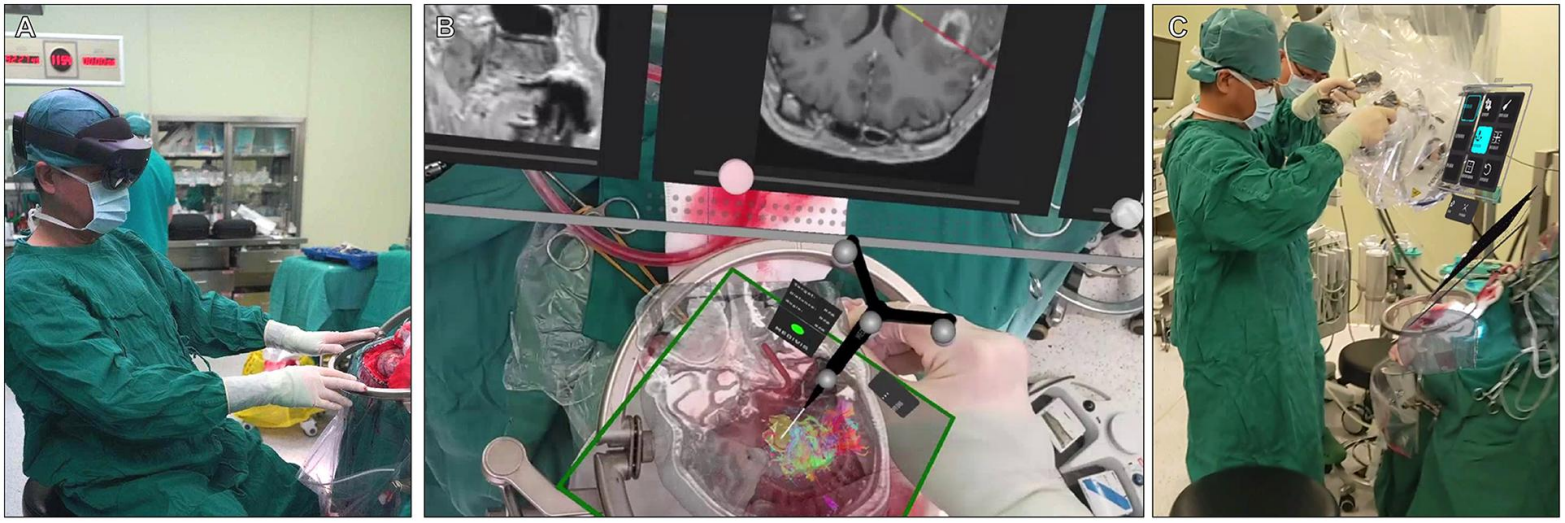
Real-time AR navigation. Upon wearing the HoloLens 2 **(A)**, the surgeon can visually locate the spatial position of the tumor and its critical adjacent structures directly within his field of view. By using a navigation probe to explore the surgical area and integrating cross-sectional imaging, the extent of lesion resection can be accurately evaluated **(B)**. During microsurgical tumor resection, the surgeon removes the HoloLens 2 and operates solely through the surgical microscope **(C)**.

During glioma resection, the optical tracking reference frame were tracked via sensors on HoloLens 2, and the position of the AR image was updated in real time. AR navigation was utilized to visualize tumor margins in real time, facilitating maximal lesion resection. Using the navigation probe, the surgeon can also view 2D axial, sagittal, and coronal trajectory projections of the surgical segment adjacent to the surgical area, as demonstrated in Figure 3.

However, with the partial resection of the tumor and the loss of cerebrospinal fluid, brain shift was visually apparent in 2 cases, reducing the alignment accuracy of deep structures. The surgeon compensated by relying on intraoperative ultrasound when mismatch was observed.

**Figure 4:**
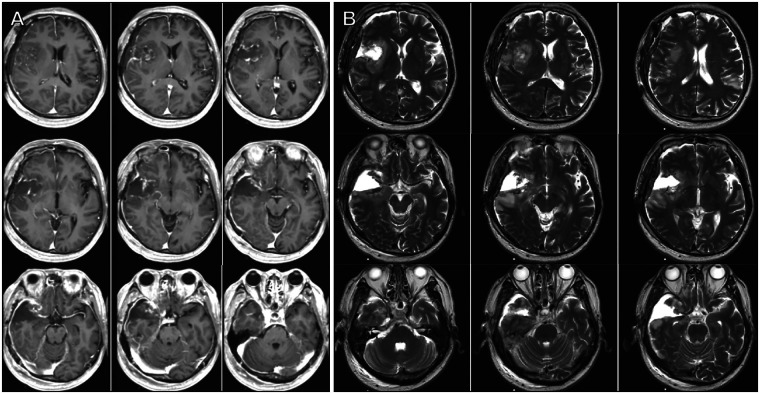
Postoperative image acquisition. Postoperative MRI at 48 h revealed complete resection of the enhanced lesion [**(A)**, T1CE], with no evidence of complications such as significant hemorrhage or cerebral infarction in the surgical area [**(B)**, T2WI].

A mean registration time of 4.7 ± 0.7 min (range 4–6 min). The TRE measured against the StealthStation landmarks averaged 2.1 ± 0.4 mm (range 1.8–2.5 mm). The holographic overlay remained stable during the initial phase of surgery. The GTR of contrast-enhancing tumor was achieved in all six patients on postoperative MRI (Figure 4).

## Discussion

This pilot study demonstrates the technical feasibility of a head-mounted AR navigation system for brain glioma resection in a small patient series. We achieved a mean registration time under 5 min and a TRE of approximately 2 mm, which is within the range reported for other AR platforms (8, 10, 12). The ability to visualize holographic tumor margins, vascular structures, and fiber tracts directly in the surgical field was subjectively helpful for surgical planning and intraoperative orientation. All 6 patients underwent gross total resection, and functional outcomes improved significantly at 3 months.

Our TRE of 2.1 mm is comparable to the 2.3 mm reported by Van Gestel et al. for AR-guided ventricular drain placement (8) and the 1.9–2.7 mm range observed in other HoloLens-based neurosurgical series (13, 15). The registration time of 4.7 min is similar to that described by Luzzi et al. (10) and suggests that the surface-matching workflow adds minimal time to the procedure. However, direct comparison is limited by differences in registration protocols and the lack of standardized accuracy metrics across studies.

Several important limitations must be acknowledged. First, the sample size of six is small and typical of a pilot feasibility study. Therefore, we cannot generalize the 100% GTR rate or the functional improvements to a broader population. Selection bias and the absence of a control group preclude any causal attribution of outcomes to the AR system. Second, the assessment of resection extent was not blinded; the operating surgeon also evaluated postoperative MRI, introducing potential detection bias. Future studies should involve independent neuroradiologists masked to the navigation method. Third, the mRS, while widely used, lacks the granularity to capture subtle neurological deficits in motor or language function. We supplemented it with structured examinations, but more sensitive scales should be incorporated in future work.

A major unresolved challenge is brain shift. With the partial resection of the tumor and the loss of cerebrospinal fluid, brain shift was visually apparent in 2 cases, reducing the alignment accuracy of deep structures. The preoperative images no longer perfectly reflect the intraoperative anatomy. In 2 of our cases, noticeable displacement of deep structures occurred, reducing the accuracy of the AR overlay. Moreover, peritumoral edema can distort DTI-derived tractography both preoperatively and intraoperatively, as highlighted by recent studies (19–22). Although we did not quantitatively assess the impact of edema on fiber tracking, we are aware that edema-associated tissue deformation may affect the reliability of the displayed tracts. In our series, the surgeon used the AR display as an initial guide but confirmed tumor locations with the intraoperative ultrasound when necessary. Future iterations could integrate intraoperative low-field MRI to update the holographic model in real time and compensate for brain shift.

We observed a trend toward shorter registration times in later cases: the first two cases required 5–6 min, while the last three were completed in 4 min. This suggests a learning curve for both the surgeon and the operating room staff. Formal analysis with more cases would be valuable to quantify the efficiency gains over time. The HoloLens 2 device used in this study has been discontinued by Microsoft, raising concerns about the long-term availability of hardware for clinical adoption. While this does not diminish the scientific value of our pilot data, it underscores the need for collaboration with medical technology companies that can provide sustained support and regulatory clearance for surgical navigation devices. Alternative AR headsets are emerging, and the software platform (SurgicalAR) may be adaptable to other hardware.

To move beyond feasibility, larger prospective studies with control groups are essential. A randomized trial comparing AR-assisted resection with conventional neuronavigation would require at least 30–40 patients per arm to detect clinically meaningful differences in extent of resection or neurological outcome. Such a trial should incorporate blinded outcome assessment, standardized accuracy metrics, and cost-effectiveness analysis. Additionally, technical improvements in non-rigid registration and integration with intraoperative imaging are needed to address brain shift.

## Conclusion

This pilot study shows that real-time AR navigation with a head-mounted display is feasible in brain glioma surgery, offering intuitive visualization and acceptable registration accuracy. The positive clinical outcomes observed in this small series are encouraging but must be interpreted with caution due to the lack of a control group and small sample size. Further technical development and rigorous comparative studies are required to establish the role of AR in routine neurosurgical practice.

## Data Availability

The original contributions presented in the study are included in the article/Supplementary Material, further inquiries can be directed to the corresponding author.

## Supplementary material

Supplementary Video 1 Real-time AR navigation during brain glioma resection.
